# Prime-boost vaccination with chimeric antigens adjuvanted in Montanide™ ISA50 V2 confers protection against experimental *Lepeophtheirus salmonis* infestation in Atlantic salmon (*Salmo salar* L.)

**DOI:** 10.3389/fimmu.2025.1570948

**Published:** 2025-05-21

**Authors:** Alianet Rodríguez, Koestan Gadan, Lincidio Pérez, Øystein Evensen, Mario Pablo Estrada, Yamila Carpio

**Affiliations:** ^1^ Animal Biotechnology Department, Center for Genetic Engineering and Biotechnology (CIGB), Havana, Cuba; ^2^ Faculty of Veterinary Medicine, Sea lice Research Centre, Norwegian University of Life Sciences, Aas, Norway

**Keywords:** chimeric antigens, *Lepeophtheirus salmonis*, prime-boost, salmon, vaccines

## Abstract

**Introduction:**

Sea lice are crustacean ectoparasites affecting Atlantic salmon production worldwide and impediments to industry growth. Chemical treatment has been the method of choice to control infestation with increasing resistance. Vaccination is an environmentally friendly alternative for sea lice control; however, obtaining high levels of lice reduction through active immunization has proven difficult. This study aimed to explore the efficacy of two sea lice vaccine prototypes under laboratory-controlled conditions.

**Methods:**

Therein, fish were vaccinated with two chimeric antigens, TT-P0 or P0-my32, using oil-adjuvanted vaccine formulations and a prime-boost vaccination protocol. Fish were experimentally challenged with copepodids at 2, 5, and 11 months post*-*prime vaccination.

**Results and discussion:**

TT-P0 vaccinated fish had a significantly lower lice number at all three challenges, 88, 90, and 20%, respectively, compared to controls. The P0-my32 vaccine gave high protection at early time points post-vaccination, with 91 and 75.4% reduction at 3 and 6 months, respectively, fading off at 12 months (4.2% reduction vs. control). The TT-P0 group had a significantly lower lice number than controls at the 11-month challenge. A higher degree of protection coincided with higher circulating antibody levels against homologous antigens. This proof of concept study encourage the use of vaccination as a tool to reduce the lice burden in salmon, and preclinical and clinical testing at a large scale is needed to document the level of protection attained under field conditions.

## 1 Introduction

Salmon aquaculture is a complex and multifaceted industry with significant environmental, economic and social implications. Norway, Chile, Scotland and Canada are the top producing countries being Atlantic salmon (*Salmo salar* L.) the most farmed salmon species. Sea lice (*Lepeophtheirus salmonis* and *Caligus* spp.) are parasitic crustaceans that attach to salmon, feeding on their skin, mucus and blood. They are one of the most costly and damaging challenges in salmon farming, affecting fish health, farm economics and wild salmon populations (1, 2).

Sea lice control in Norway is based on weekly monitoring of lice numbers in 20 fish in all cages of each farm, with mandatory delousing initiated when louse levels exceed allowable limits, which vary over the year. The introduction of chemical treatments in the ‘90s allowed farms to treat sea louse infestations without substantially reducing production (3). However, most chemotherapeutants have an environmental impact, and concerns have been raised about bioaccumulation and its effects on non-target invertebrate species. Further, treatment resistance has emerged, reducing the efficacy of various chemical treatments (2–4). The appearance of treatment resistance has shifted producers toward non-medicinal delousing in Norway (5), and these methods are also gaining attention in other salmon-producing countries. Examples of these procedures include bathing salmon in heated water (up to 32°C) for 30 seconds or mechanical high-pressure water flushing to remove lice from the fish (5, 6), imposing stress and consequently impacting animal welfare (5). Many producers also co-cultivate various species of cleaner fish with the salmon (7, 8) since cleaner fish feed on salmon lice. Still, the mortality rate among cleaner fish is high, which raises ethical concerns, and cleaner fish can also be a source of disease for the salmon. Another method employed is the laser elimination of adult lice (9). In this context, an effective sea lice vaccine would be of great interest and represent a prophylactic tool rather than therapeutic. However, the development of vaccines against ectoparasites is still technically challenging. Despite identifying several vaccine targets in a range of ectoparasites, the Gavac vaccine against the cattle tick (*Rhipicephalus microplus*) remains the only effective ectoparasite vaccine (10, 11).

Akirin and subolesin are evolutionarily conserved orthologous transcription factors involved in regulating the expression of signal transduction and innate immune response genes in vertebrates and invertebrates (12). Akirin/Subolesin is a well-characterized antigen that has been proposed for the development of a broad-spectrum vaccine for the control of arthropod infestations (13). We have characterized this gene, denoted as my32, from *C. rogercresseyi* and *L. salmonis* (14, 15). Additionally, it has been shown that immunization with recombinant versions of this protein produced in *Escherichia coli* reduced *C. rogercresseyi* infestations in a vaccine trial in Atlantic salmon when lice counts were performed at the second parasite generation (14). In addition, promiscuous T-cell epitopes from tetanus toxin and measles virus (TT) conjugated to the N-terminus of a 35 amino acid peptide from the ribosomal P0 protein of *L. salmonis* were shown to elicit significantly higher humoral immune responses after immunization (16), and with a significant reduction in adult female lice in vaccinated fish compared to controls (17).

The present study compared the chimeric P0-my32 protein with the P0 peptide fused to the molecular adjuvant TT regarding immunogenicity and the ability to reduce lice infestation in Atlantic salmon after the experimental challenge. Both proteins, P0-my32 and TT-P0, were produced in *E. coli*, purified, and formulated with Montanide™ ISA50 V2 as an adjuvant. A prime-boost vaccination was used and challenges were performed at 2-, 5- and 11-months post-vaccination to assess the onset of immunity and the duration of protection. These results suggest that vaccination can reduce lice infestations in Atlantic salmon, but additional studies are needed to confirm efficacy under field conditions.

## 2 Materials and methods

### 2.1 Transcription profiling by RT-PCR

Different developmental stages of *L. salmonis* were sampled from anaesthetized fish [nauplius and copepodids (2 pools of n∼1000 each), chalimus (2 pools of n∼100), and males and females (2 pools of n∼50 each)]. Samples were kept in RNA-later (Ambion) at -80°C until use. First, the RNA-later solution was discarded and the Tri-reagent (Promega) solution was added. Afterward, the tissue in the Tri-reagent solution was homogenized using mortar and pestle and RNA isolation was performed according to the manufacturer’s instructions. The measurement of UV absorbance at 260 nm, A260/A280 ratio and denaturing gel electrophoresis was done to check RNA concentration and quality. Genomic DNA was removed by DNase I digestion (Promega).

The P0 transcription profile was determined by RT-PCR using actin as a reference gene. Specific-gene primers (**Table 1**) were designed according to the *L. salmonis* P0 (BT077866) (primers A and B) and β actin (EF490906.1) sequences (primers C and D). PCR Master Mix (M7502, Promega) was used for amplifications according to the manufacturer’s instructions. In these reactions, 2 µl of neat cDNA samples were used as templates. Each primer was used at a final concentration of 20 pM. The reactions were set at 25 µL final volume. PCR amplifications were performed under the following conditions: initial denaturation at 94°C for 2 min, 35 amplification cycles (denaturing at 94°C for 30 s, annealing temperature at 58°C, and extension at 72°C for 1 min), and a final extension step at 72°C for 5 min. Three technical replicates were done for each developmental stage. The PCR products were separated by 2% agarose gel electrophoresis. Control reactions were performed using the same procedures but without adding the reverse transcriptase enzyme as a control for DNA contamination in the RNA preparations and without cDNA added as a control for contamination of the PCR reaction.

**Table 1:** Primers used in gene isolation, gene expression studies, and recombinant *Escherichia coli* expression.

Primer name	Gene	Genebank Accession number	Sequence	Direction
A	*L. salmonis* P0	BT077866	GATGAAGCCCAATCCAAGAGAG	Forward
B	*L. salmonis* P0	BT077866	AGGCATAGAGGGAGAGGACAG	Reverse
C	*L. salmonis* β actin	EF490906.1	CGACGAGTACCCCAAGTGTT	Forward
D	*L. salmonis* β actin	EF490906.1	ACCCAAGCCTGTGTTTTGAG	Reverse
E	*L. salmonis* P0	BT077866	CCATGGAATATCTGGCTGATCCCA	Forward
F	*L. salmonis* P0	BT077866	GGATCCCTCAGGTTCATCCGCCTTAG	Reverse
G	*L. salmonis* my32	ADD38399	GGATCCGCTTGTGTTACTCTTAAACGTCC	Forward
H	*L. salmonis* my32	ADD38399	AAGCTTATAACTCGGAGTTTGGGAGTC	Reverse

Endonuclease restriction sites are underlined.

### 2.2 Cloning and expression in *Escherichia coli* of P0-my32 and TT-P0 antigens

To obtain the chimeric protein P0-my32, the sequence coding for the 35 aa P0 peptide between the amino acids 267 to 301 from *L. salmonis* P0 was amplified first. As template, P0 cDNA previously isolated in the laboratory and already cloned in pMOS-Blue (GE Healthcare) was used (16). This sequence was amplified using the primers E and F containing the restriction sites *Nco* I-*Bam*H I (**Table 1**) to allow fusion to the N-terminal of the my32-Ls antigen (15). This PCR fragment was sub-cloned into pGEM-T-easy (Promega) following manufacturer´s instructions. Once the sequence of this P0 fragment with the proper restriction sites was confirmed, the band was extracted by endonuclease digestion (*Nco* I-*Bam*H I) and inserted into the same cloning sites of pET28a. Afterward, my32 was amplified with primers G and H containing BamH I-Hind III restriction sites (**Table 1**) using pMOS-Blue-my32-Ls (15) as template to allow my32-Ls insertion at the C-terminal of P0 peptide in the pET28 expression vector. In this construct, the inserted gene was controlled by the inducible T7 promoter and yielded a polypeptide with a fused C-terminal histidine tail. The pET28a-P0-my32 expression plasmid was transformed into *Escherichia coli* BL21 (DE3) strain for the recombinant polypeptide expression. Leal et al. described the cloning of TT-P0 antigen (16).

Both P0-my32 and TT-P0 antigens were produced by auto-induction during fermentation. The inoculum for fermentation was a flask containing 350 mL of non-inducible medium previously inoculated with 100 μL of 20% glycerol stocks and grown for 16 h at 37°C in a shaker incubator. This non-inducible medium was composed of 3.5 mL of the 50X M medium (1.25 M Na_2_HPO_4_, 1.25 M KH_2_PO_4_, 2.5 M NH_4_Cl, 0.567 M Na_2_SO_4_); 0.25% (w/v) glucose; 0.2% (w/v) L-aspartic acid; 1 mM MgSO_4_·7H_2_O, 0.1 mM FeCl_3_-CaCl_2_ and 100 μg/mL kanamycin. Complex medium II (CM II), modified from Studier (18) was used in the fermentation process: 20 mL of the 50X M medium per liter, 20 mL of the 50X 5052 medium per liter (44.4% glycerol (w/v), 0.139 M glucose, 0.277 M α-lactose monohydrate), 2 mM MgSO_4_·7H2O, 0.1 mM FeCl_3_-CaCl_2_, 10 g/L tryptone, 5 g/L yeast extract, 10 g/L NaCl, 100 μg/mL kanamycin. Lactose was used as an inducer in an auto-inducing procedure for the CM II medium. The culture was grown for 7 or 10 h in a 5-L INFORS HT bioreactor (Switzerland) containing 3.5 L at 37°C, pH=7, and 600 rpm agitation. Aeration was set at 1 vvm. Foam formation was avoided by adding Antifoam Glanapon DG 158 (Bussetti, Austria).

The cell culture was centrifuged at 10 000 x g for 10 min at 4°C. The cell pellet was suspended in lysis buffer (300 mM NaCl, ten mM Tris, pH 6) and disrupted in a model APV Gaulin high-pressure homogenizer (2-Stage 15MR8TBA 5E8870)) at 600–800 kgf/cm^2^. The disrupted cell suspension was centrifuged at 10 000 x g for 10 min at 4°C. The insoluble fraction containing the recombinant proteins as inclusion bodies was suspended in 1M NaCl, 1% Triton X-100 using polytron Ultra-Turrax T25, IKA WERKE, and centrifuged again at 10 000 x g for 10 min at 4°C.

### 2.3 Purification of P0-my32 and TT-P0 antigens

For purification of P0-my32 and TT-P0 antigens, the insoluble fraction containing the recombinant proteins was suspended in solubilization buffer (100 mM NaH_2_PO_4_, 10 mM Tris, 10 mM Imidazole, 8 M urea, pH 8). It was incubated for 2 h at 37°C with gentle agitation. Afterwards, the sample was centrifuged at 10 000 x g for 20 min at 4°C and the supernatant was used for further purification steps. Affinity chromatography was performed under denaturing conditions employing IMAC Sepharose™ Fast Flow (GE Healthcare) according to the manufacturer’s instructions mounted on an AKTA-Pure system (GE Healthcare, Piscataway, NJ, USA). The clarified lysate with 10 mM imidazole was loaded onto the previously equilibrated column with equilibration buffer (100 mM NaH_2_PO_4_, Tris 10 mM, imidazole 10 mM, urea 8 M, pH 8). Then, wash and elution were performed with the same buffer but with 40 mM and 150 mM imidazole, respectively. The eluted fraction containing the purified proteins was loaded onto a G-25 (GE Healthcare) column equilibrated with the vehicle buffer (100 mM NaH_2_PO_4_, 10 mM Tris HCl, 0.1% Tween-20) for refolding. Recombinant proteins were concentrated using an Amicon Ultra-15 ultrafiltration device (cut off 3 kDa) (Millipore-Merck, Darmstadt, Germany).

According to the manufacturer’s instructions, protein concentration was determined with a Bradford protein assay kit (Pierce). The purity of recombinant proteins was assayed by densitometry scanning of protein gels considering total protein concentration.

### 2.4 Protein gel electrophoresis and western blotting

SDS-PAGE and immunoblotting confirmed the presence of the recombinant protein. Protein samples were loaded on 15% polyacrylamide gels under reducing conditions and stained with Coomassie Brilliant Blue. For western blotting, the proteins separated on the gel were transferred to a nitrocellulose membrane (GE Healthcare, Amersham, Germany). Membranes were blocked with 5% skim milk for 60 min at room temperature. After washing with phosphate-buffered saline (PBS)-Tween 0.01% once and with PBS twice, the membrane was incubated with an anti-His monoclonal antibody peroxidase conjugate (A7058, Sigma) at a dilution 1:2–000 for 60 min at room temperature. After the washing steps, chromogenic detection was carried out using an ECL detection system.

### 2.5 In-gel protein digestion and mass spectrometry analysis

The identity of the purified protein was confirmed by mass spectrometry analysis. The Coomassie blue-stained band was excised from SDS-PAGE gels and incubated at 37°C with 50% acetonitrile in 1% ammonium bicarbonate until colorless. The gel slice was dried and rehydrated in 25 mM ammonium bicarbonate buffer containing sequencing-grade trypsin at 12.5 ng/μL. The in-gel digestion was for 16 h at 37°C. The resulting proteolytic peptides were passively eluted in 0.2% formic acid solution, desalted using a ZipTips reverse phase micro column, and loaded into gold-coated borosilicate nanotips for mass spectrometry analysis.

Low-energy ESI-MS and MS/MS spectra were acquired using a QTOF-2™ mass spectrometer from Waters (Manchester, UK). The capillary and cone voltages were set to 1200 and 35 V, respectively. The multiply-charged signals of the highest intensity corresponding to tryptic peptides were further analyzed by ESI-MS/MS using appropriate collision energies to obtain partial or complete amino acid sequences.

### 2.6 Vaccine formulation

Purified recombinant proteins TT-P0 and P0-my32 were formulated in Montanide™ ISA50 V2 (Seppic, Paris, France) at a ratio of 50/50 (w/w). Formulations were made using a Politron (Ultra-Turrax T25, IKA WERKE, Germany).

### 2.7 Immunogenicity in mice

Mice studies were approved by the Ethical Committee on Animal Experimentation of the Center for Genetic Engineering and Biotechnology (CIGB, Havana, Cuba). Female BALB/c of 5–6 weeks and 15–18 g were used. Ten mice per group were immunized by intraperitoneal injection with P0-my32 or TT-P0 vaccine formulations at a dose of 20 µg per mouse in a total volume of 20 µL on days 0 and 14. Control mice were immunized with the same volume of adjuvanted vehicle buffer. Blood was collected by retro-orbital bleeding 28 after the beginning of the experiment (14 days after booster). Serum for antibody analysis was obtained by centrifugation at 10 000 x g for 5 min. Antigen-specific total IgG antibodies in the sera of vaccinated mice were determined by a sandwich ELISA. High-binding microtiter plates (Costar) were coated overnight, using 5 µg/mL of purified protein. After blocking with 3% skim milk for 1 h at 37°C, two-fold serial dilutions of sera were applied from 1:500 to 1:32–000 and incubated for 2 h at 37°C. After 4 wash steps with PBS-Tween 0.05%, bound antigen-specific antibodies were detected by incubation with anti-mouse IgG (Sigma-Aldrich, USA) conjugated with peroxidase at 1:10 000. Followed by 4 wash steps, the addition of substrate solution developed the reaction. After 10 min, the reaction was stopped with a stop solution. The color intensity was measured at 450 nm. The titer was the highest dilution, giving an optical density twice the value of the pre-immune serum.

### 2.8 Salmon vaccination and challenge trial

#### 2.8.1 Fish

Postsmolt of Atlantic salmon (*Salmo salar*), strain AquaGen, with 50–60 g at the time of first vaccination – were used. Sexually matured, injured or deformed fish were excluded from the study during the experiment. The trial was performed according to the Standard Operating Procedures at Solbergstrand/NIVA, Norway. Fish were marked with pit tags and they were acclimatized for 14 days after transfer to the experimental facility. The fish and tanks were fed and monitored daily. Mortalities were also collected daily. Intake water temperature was observed. Environmental and handling parameters are summarized in **Supplementary Table S1**. The experiment was approved by the Food Inspection Agency, FOTS ID 19688, Norway.

According to Solbergstrand standards, the fish were fed with an automatic feeder (feeding of periods of 20 sec 5 times per day). Each day before and after challenge feeding, the target feeding level was 1-2% body weight/day, with an average ingestion rate of 1.5% of fish body weight/day.

#### 2.8.2 Fish vaccination

For fish vaccination experiment, 300 Atlantic salmon were randomly partitioned into four experimental groups: three test groups and a non-vaccinated control group. The trial included three parallel tanks per experimental group with 25 fish per tank (n=75 fish per group). Fish were anesthetized with benzocaine 20% (Euro-pharma) and vaccinated by intraperitoneal injection with 0.1 mL of TT-P0 vaccine formulation (100 µg/0.1 mL of TT-P0 antigen), P0-my32 vaccine formulation (100 µg/0.1 mL of P0-my32 antigen) or vehicle buffer adjuvanted in Montanide ISA50 V2 (adjuvant control group). Vaccination was done manually. The time of the first vaccination was set as study week 1. At 350 degree days (DD) post prime injection, a booster dose was administered using the same method as the first injection. Afterward, the fish were gradually acclimatized to seawater. The timeline for the experiment is summarized in **Table 2**.

**Table 2:** Timeline for salmon vaccination experiment using P0-my32 and TT-P0 as antigens and challenge with *Lepeophtheirus salmonis*.

Activity	Study week	Comments
Weigh fish, transfer into trial tank		Good quality fish
Pre-feeding period		Good growth Kept in recirculation system
Vaccination-Prime	Week 1 (First injection defines time zero)	–
Vaccination-Boost	Week 4 (Boost at 350 degree days)	–
Transfer to challenge facility	Week 7	Transfer in plastic bags, no mortalities
Transfer to sea water	Week 7	Turned on sea water, flow through system
Challenge with copepodids	Week 7	300 degree days after booster
First counting of sea lice	Week 9	12 days (chalimus stage) post challenge. Counting of 9 fish/group.
Second counting of sea lice	Week 12	32 days post challenge.All fish counted.
Deloused with freshwater	Week 18	
2nd infection with copepodids	Week 19	New batch of copepodids, check for activity and control counting performed
Examined for success of infection	Week 22	Brief examination of fish, no counting. Small lice/early development stage.
Counting of 2^nd^ challenge	Week 25	All lice at preadult/adult No mature males or females.
Delousing	Week 26	–
3rd challenge	Week 44	–
Counting 3rd challenge	Week 51	Preadult/adult lice stage

#### 2.8.3 Challenge trials with *Lepeophtheirus salmonis*

The first challenge with sea lice (*L. salmonis*) copepodids was 300 DD post-booster immunization. Copepodids were obtained from Industry Laboratory, Norway. Fish were exposed to 30 copepodids/fish (17) for 30 min in stagnant water while oxygen was added to keep concentration >80% saturation. Water flow was resumed when the challenge period was completed (19).

Counting was performed 12 and 32 days post-challenge (dpc). Counting at 12 dpc was done in 9 fish from each group (3 fish randomly picked from the 3 parallel tanks per group). At 32 dpc, the total number of lice on each fish was counted and the different stages were determined. Fish were treated with freshwater after counting to ensure no lice were present before next challenge. The second and third challenges with new copepodids were done in study weeks 19 and 44. Counting was done in study weeks 25 and 51, respectively (**Table 2**).

The second challenge was performed in three replicate tanks per group using the above mentioned protocol. The third challenge was a common garden experiment with fish from all vaccine groups kept together. After the third challenge, fish were euthanized and side-effect scores were registered.

#### 2.8.4 Side effects

After lice counting, injection site reactions in the peritoneal cavity were recorded and scored as described (20). The fish were euthanized by cranial concussion and the peritoneal cavity was opened by a ventral incision for inspection. The assessment of side effects included the inspection for adhesions between the parietal wall and the internal organs and/or adhesions between organs. Melanin deposition was recorded at the site of injection and in the visceral peritoneum (over the internal organs). Fish were weighed before opening the abdominal cavity.

#### 2.8.5 ELISA tests

Plasma was collected from 12 fish in each treatment group (TT-P0, P0-my32, and adjuvant control) and non-injected (control) fish at the final counting of the 3^rd^ challenge. After being put in deep anesthesia, blood was collected from the caudal vein using vacutainers (heparin as anticoagulant) and transferred to ice before centrifuging (3 500 × g, 10 min at 4°C). The purified proteins were used to coat the ELISA plates (1 µg/mL of protein) overnight at room temperature. The following day, plates were washed (3x) with PBS at room temperature. Plasma samples were diluted 1: 400 in sterile water and incubated at room temperature for 30 min, followed by 3x washing with PBS. The reaction was developed with a peroxidase-conjugated monoclonal antibody, anti-IgM (diluted 1: 3 000), followed by 3x washing in PBS and then adding 100 µl substrate solution. This was followed by incubation for 15 min, after which a stop solution (0.01M H_2_SO_4_) was added. The color was read at 492 nm.

### 2.9 Statistical analysis

Statistical analysis and graphs were made using the Prism 8.02 software for Windows (GraphPad Software, San Diego, CA, USA) or Stata 16, College Station, TX 77845, USA. Power analysis was conducted to determine optimal sample size in the fish experiment using GPower (21). Lice numbers between treatment groups were compared using Poisson regression or Negative binomial regression analysis, pending the distribution of lice in the test groups (Stata 16). Differences in adhesions and antibody levels among groups were analyzed with a non-parametric statistical method (Kruskal-Wallis equality-of-populations rank test).

## 3 Results

### 3.1 P0 gene expression analysis in *L. salmonis* life cycle, production of P0-my32 and TT-P0 recombinant proteins and immunogenicity in mice

Gene expression analysis showed that P0 mRNA is detected in all the developmental stages assayed: nauplius, copepodids, chalimus, and adults. The β actin amplification corroborated the quality of the cDNA samples (**Supplementary Figure S1**).

**Figure 1:**
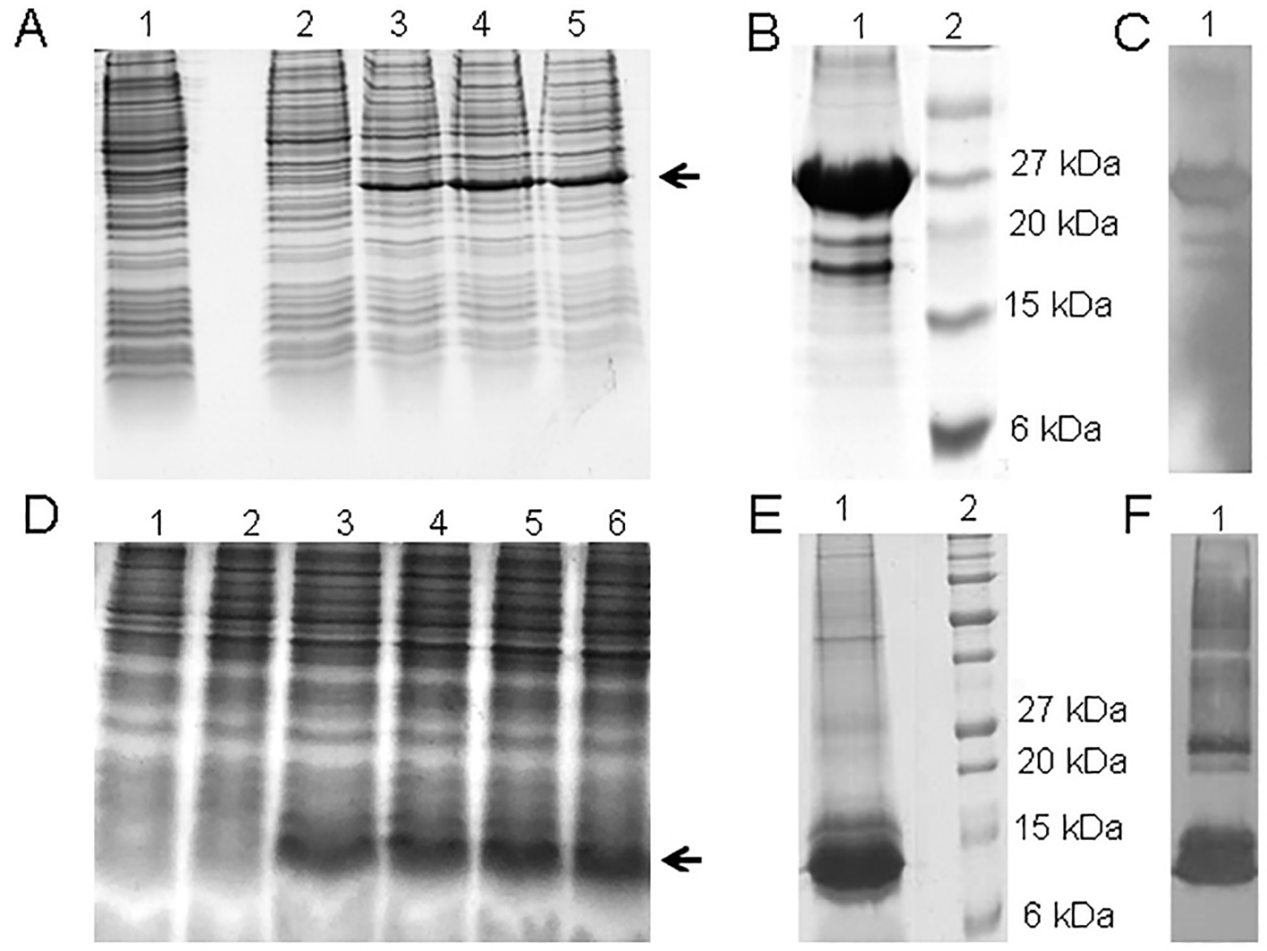
**(A)** SDS-PAGE 15% of 3.5 L P0-my32 fermentation process. Lanes 1-5: 0, 2, 4, 6, and 7 h of fermentation. The arrow indicates the target protein. **(B)** 15% SDS-PAGE and **(C)** Western blot with anti-His monoclonal antibody of the final product after P0-my32 purification and buffer ex-change. Lane 1: P0-my32, lane 2: Molecular weight marker. **(D)** SDS-PAGE 15% of 3.5 L TT-P0 fermentation processes. Lanes 1-6: 0, 2, 4, 6, 8 and 10 h of fermentation. **(E)** 15% SDS-PAGE and **(F)** Western blot with anti-His monoclonal antibody of the final product after TT-P0 purification and buffer exchange. Lane 1: TT-P0, lane 2: Molecular weight marker.

The recombinant protein expression analysis of chimeric P0-my32 showed a band between 20 and 28 kDa in the lane corresponding to BL21(DE3)-pET28a-P0-my32 *E. coli* cell extracts after 4 to 7 hours of fermentation (**Figure 1**). A wet biomass of 40 g was obtained at the end of the fermentation process. The estimated size based on amino acid sequence was approximately 25 kDa. After cell disruption, most of the protein was obtained forming inclusion bodies. The protein was obtained with 86% purity (**Figure 1**) after solubilization in urea and purification by affinity chromatography to niquel metal chelates. The presence of the histidine tag was confirmed using an anti-His monoclonal antibody (**Figure 1**).

The expression analysis of the TT-P0 protein showed a band between 6 and 15 kDa in the lane corresponding to BL21(DE3)-pET28a-TT-P0 *E. coli* cell extracts after 4 to 10 hours of fermentation (**Figure 1**) and 63 g of wet biomass was obtained at the end of the fermentation process. The obtained band agrees with the expected molecular weight for the TT-P0 protein of 8.3 kDa, according to the prediction based on the amino acid sequence deduced from the nucleotide sequence. After purification, the protein was obtained with 91% purity (**Figure 1**). The western blotting result also confirmed the presence of the His-tag (**Figure 1**).

ESI-MS/MS analyses of the peptides derived from the tryptic digestion of the purified P0-my32 were performed. Spectra were manually analyzed, and reliably extracted sequences were used to confirm the identity of the recombinant P0-my32 (**Supplementary Figure S2**). Additionally, the mass spectrum of TT-P0 tryptic digestion of the most intense band showed few signals, typical of a low molecular weight protein and multiple tryptic sites. The most intense signals were fragmented, and the MS/MS spectra were analyzed manually. The sequences obtained confirmed the identity of the TT-P0 chimeric protein (**Supplementary Figure S3**).

The immunogenicity of both vaccine candidates was tested in mice before the salmon experiment and assessed for ability to induce antibody responses. The response was evaluated 28 days after the beginning of the experiment. The P0-my32 antigen resulted in antibody titers ranging from 8–000 to 32 000 (**Supplementary Figure S4**). At the same time, for TT-P0, all mice had specific antibody titers above or equal to 32 000, except for one mouse with a titer of 16 000 (**Supplementary Figure S4**).

### 3.2 First and second challenge in salmon

**Figure 2:**
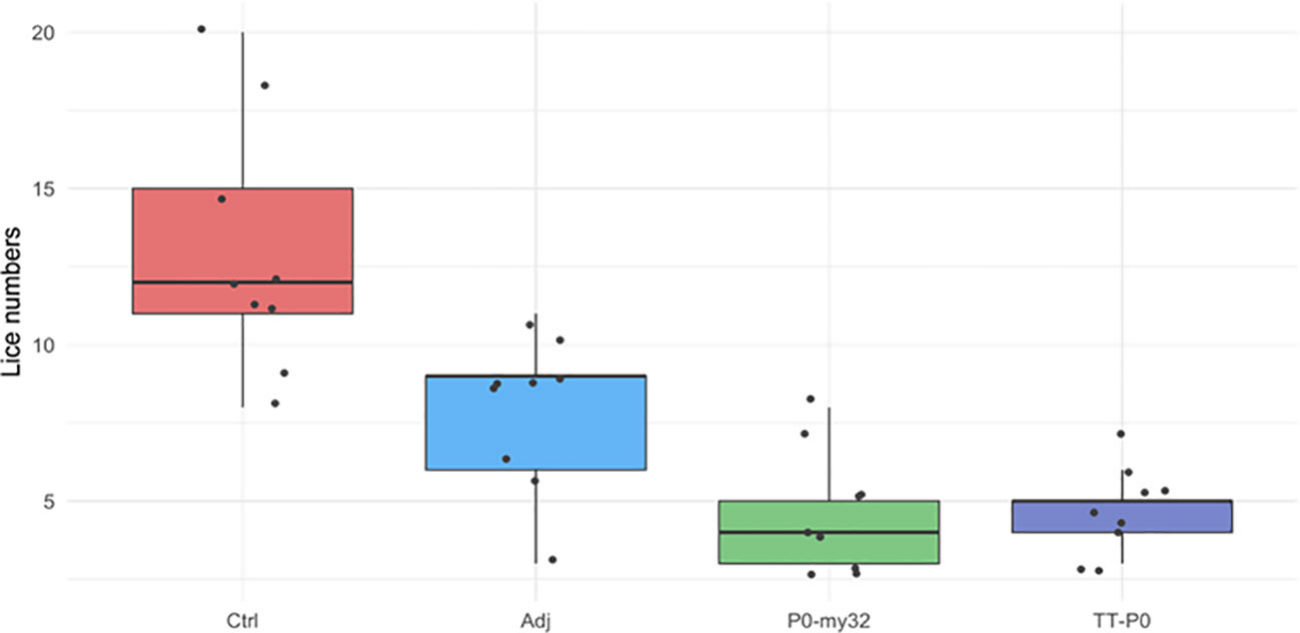
Sea lice count on individual fish (N=9 fish/group; 3 fish/tank) vaccinated with P0-my32 or TT-P0 vaccine formulations and controls. Challenge was done at 650 DD after first immunization and. lice counts (chalimus) were done 12 days after challenge. Data are presented as 25/75 quartiles and 95% CI (spikes). The black dots represent the sea lice count in each individual fish. Treatments are differentiated by colors. Ctrl: non-injected fish; Adj: injected with vehicle buffer formulated in Montanide ISA50 V2; P0-my32: Fish were injected with 0.1 mL of P0-my32 vaccine formulation (equivalent to 100 μg of P0-my32 antigen); TT-P0: Fish were injected with 0.1 mL of TT-P0 vaccine formulation (equivalent to 100 μg of TT-P0 antigen).

At 12 dpc, three fish were sampled from each replicate tank, for nine fish per experimental group. The average number of lice (chalimus) per fish in TT-P0, P0-my32, the Adjuvant-control group, and the non-injected control group was 4.7, 4.7, 8, and 12.9, respectively (**Figure 2**).

**Figure 3:**
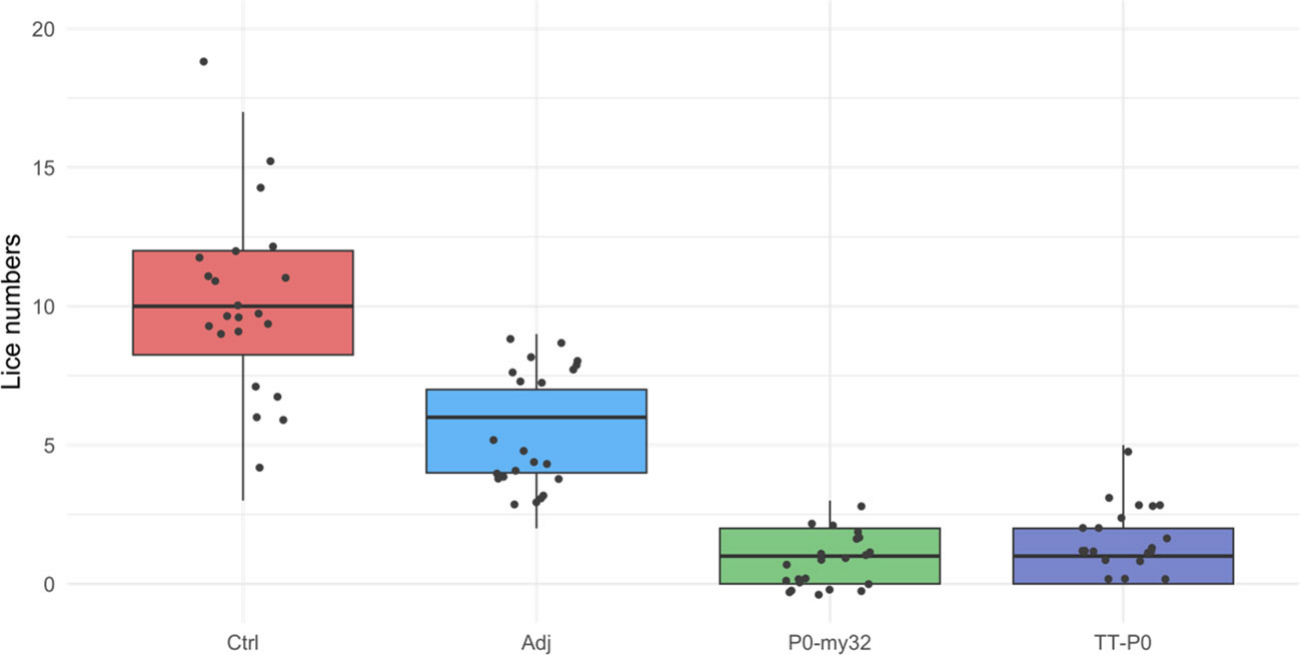
Sea lice count on individual fishes (N=74–75 fish/group) vaccinated with P0-my32 or TT-P0 vaccine formulations and controls. Challenge was done at 650 DD after first immunization. Sea lice counts were done 32 days after challenge. Data are presented as 25/75 quartiles and 95% CI (spikes). The dots represent the sea lice count in each individual fish. Treatments differentiated by colors. Ctrl: non-injected fish; Adj: injected with vehicle buffer formulated in Montanide ISA50 V2; P0-my32: injected with 0.1 mL of P0-my32 vaccine formulation (equivalent to 100 μg of P0-my32 antigen); TT-P0: Fish were injected with 0.1 mL of TT-P0 vaccine formulation (equivalent to 100 μg of TT-P0 antigen).

At 32 dpc, the lice had reached the pre-adult stage with an average number of lice in the control group of 10.5 ± 0.42 (S.E.M) lice/fish and 5.6 ± 0.24 (S.E.M) lice/fish in the adjuvant control. The P0-my32 group had an average of 0.91 ± 0.1 (S.E.M) lice/fish and TT-P0 1.3 ± 0.13 (S.E.M) lice/fish (**Figure 3**). Lice numbers are right skewed for TT-P0 and P0-my32 groups while more normally distributed for control and adjuvant control (**Supplementary Figure S5**). The variation in mean lice number between tanks within groups was < 10%. The percentage reduction was 88% and 91% in the TT-P0 group and P0-my32 group, respectively, compared to the non-injected control (p<0.0001, Poisson regression). The reduction percentage in TT-P0 and P0-my32 compared to adjuvant control was 77% and 84%, respectively. The number of lice in the adjuvant control group was reduced by 47% compared to non-injected control (p<0.0001, Poisson regression). No mortalities were recorded at this time except for one fish that was euthanized in one of the non-injected control tanks due to a skin ulcer.

**Figure 4:**
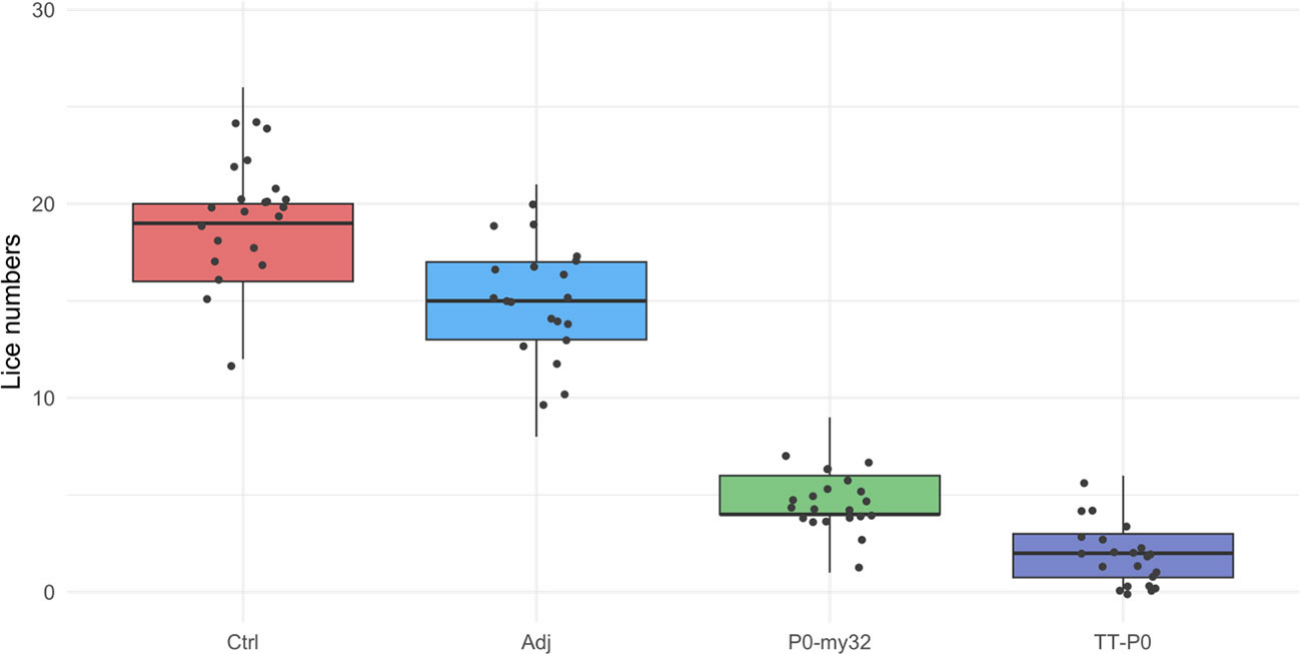
Sea lice count on individual fishes (N=64–73 fish/group) vaccinated with P0-my32 or TT-P0 vaccine formulations and controls. Second challenge was done at study week 19. Sea lice counts were done 40 days after challenge. Data are presented as 25/75 quartiles and 95% CI (spikes). The dots represent the sea lice count in each individual fish. Treatments differentiated by colors. Ctrl: non-injected fish, Adj: injected with vehicle buffer formulated in Montanide ISA50 V2; P0-my32: injected with 0.1 mL of P0-my32 vaccine formulation (equivalent to 100 μg of P0-my32 antigen); TT-P0: Fish were injected with 0.1 mL of TT-P0 vaccine formulation (equivalent to 100 μg of TT-P0 antigen).

The 2nd challenge was done 15 weeks after the boost injection. Counting of lice was carried 40 dpc, when the lice had developed to pre-adult stage with a few mature lice (< 5%). The average number of lice in the controls was 18.6 ± 0.4 (S.E.M) lice/fish (average for 3 tanks) and 14.7 ± 0.38 (S.E.M) lice/fish in the adjuvant control (**Figure 4**). The lice numbers were close to normally distributed by groups (**Supplementary Figure S6**). The TT-P0 group had 1.8 ± 0.16 (S.E.M) lice/fish, giving a 90.3% reduction relative to controls, and 4.6 ± 0.22 (S.E.M) lice/fish in the P0-my32 group, showing a decrease of 75.4% compared to the control fish (p<0.0001, Poisson regression). Further, an 88% reduction in the TT-P0 and 69.3% in the P0-my32 groups was observed compared to the adjuvant control (p<0.0001). Differences were also observed between TT-P0 and P0-my32 (p<0.001) and between adjuvant and non-injected control groups (p=0.001, Kruskal-Wallis test) (**Figure 4**). The survival per experimental group was 97%, 85%, 95%, and 96% for non-injected control, adjuvant control, P0-my32, and TT-P0, respectively.

### 3.3 Third challenge, evaluation of side effects and antibody response

**Figure 5:**
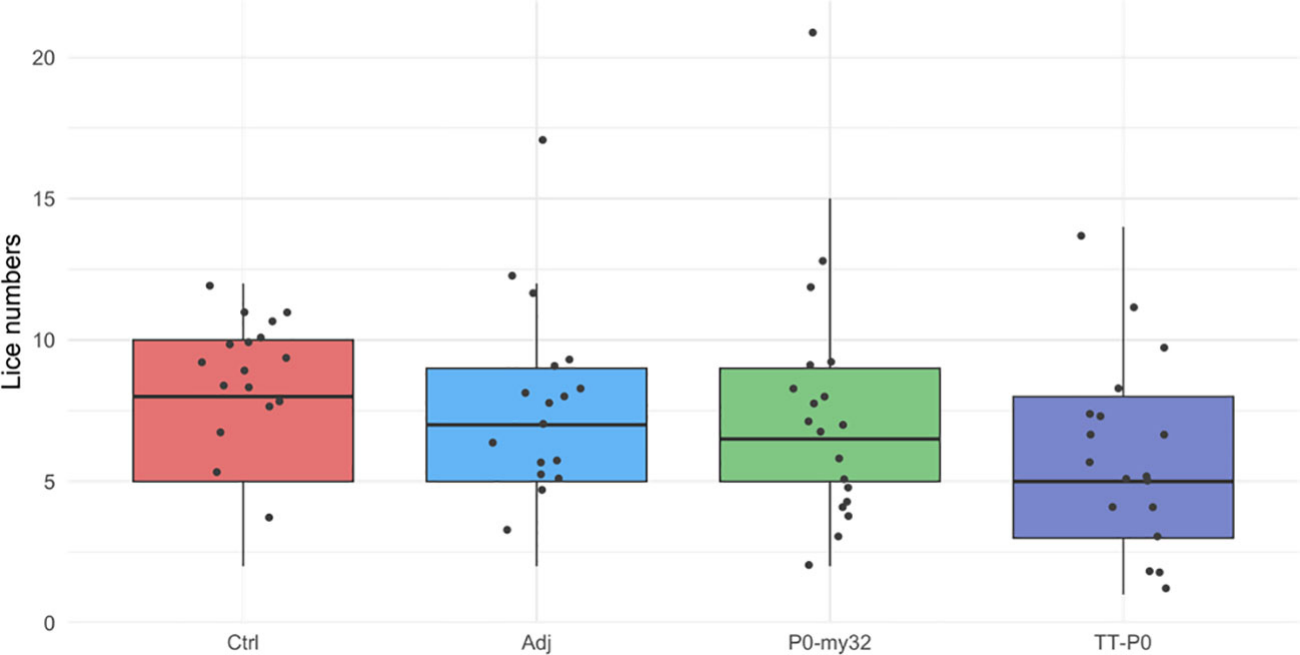
Sea lice count on individual fishes (N=52–60 fish/group) vaccinated with P0-my32 or TT-P0 vaccine formulations and controls. Third challenge was done at study week 44. Sea lice counts were done 32 days after challenge. Data are presented as 25/75 quartiles and 95% CI (spikes). The dots represent the sea lice count in each individual fish. Treatments differentiated by colors. Ctrl: non-injected fish; Adj ctrl: injected with vehicle buffer formulated in Montanide ISA50 V2; P0-my32: injected with 0.1 mL of P0-my32 vaccine formulation (equivalent to 100 μg of P0-my32 antigen); TT-P0: Fish were injected with 0.1 mL of TT-P0 vaccine formulation (equivalent to 100 μg of TT-P0 antigen).

At the end of the second challenge, the fish were deloused and transferred to a large holding tank, where all fish were kept in a standard tank up to the challenge. The third challenge commenced in week 44, which corresponded to 11 months post-boosted vaccination. Counting was carried out at experimental week 51. The results show a 20% reduction in lice numbers for TT-P0 relative to Ctrl (p=0.005, Poisson regression), a 15.8% reduction in lice numbers for TT-P0 compared to P0-my32 (p=0.04), and a 15.1% relative to Adj-ctrl (p=0.05, **Figure 5**). The survival per experimental group was 79%, 69%, 80%, and 78% for non-injected control, adjuvant control, P0-my32, and TT-P0, respectively.

**Figure 6:**
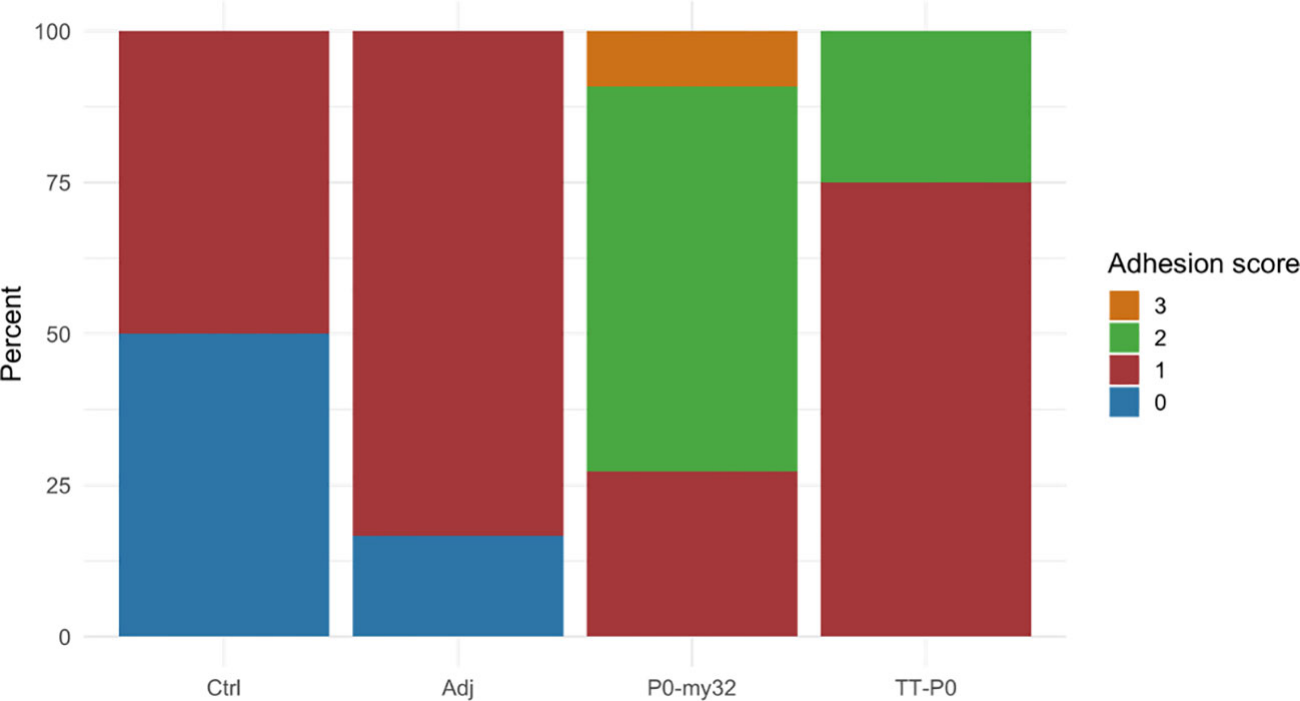
Adhesion scores for the different groups were recorded at 12 months post-vaccination. Score categories are 0–3 and y-axis indicates percentage of scores within each category. Ctrl: non-injected fish; Adj: injected with vehicle buffer formulated in Montanide ISA50 V2; P0-my32: injected with 0.1 mL of P0-my32 vaccine formulation (equivalent to 100 µg of P0-my32 antigen); TT-P0: Fish were injected with 0.1 mL of TT-P0 vaccine formulation (equivalent to 100 µg of TT-P0 antigen).

The adhesion scores for the P0-my32 vaccinated fish were higher than for the other groups (**Figure 6**, p<0.01, Kruskal Wallis test). Further, the TT-P0 group scored significantly higher than adjuvant control (p=0.0038) and non-injected control (p=0.028). Melanin scores were low for all groups, with a few fish scoring 3 in the TT-P0 group (**Supplementary Figure S7**). There was no significant difference in weight between the groups, but fish in the P0-my32 group had the lowest average weight (370.2 ± 28.4 g (S.E.M)) versus controls at 409.7 ± 30.7 (S.E.M) g. Adj ctrl was 418.6 ± 36.4 (S.E.M) g and TT-P0 was 403.2 ± 23.2 (S.E.M) g.

**Figure 7:**
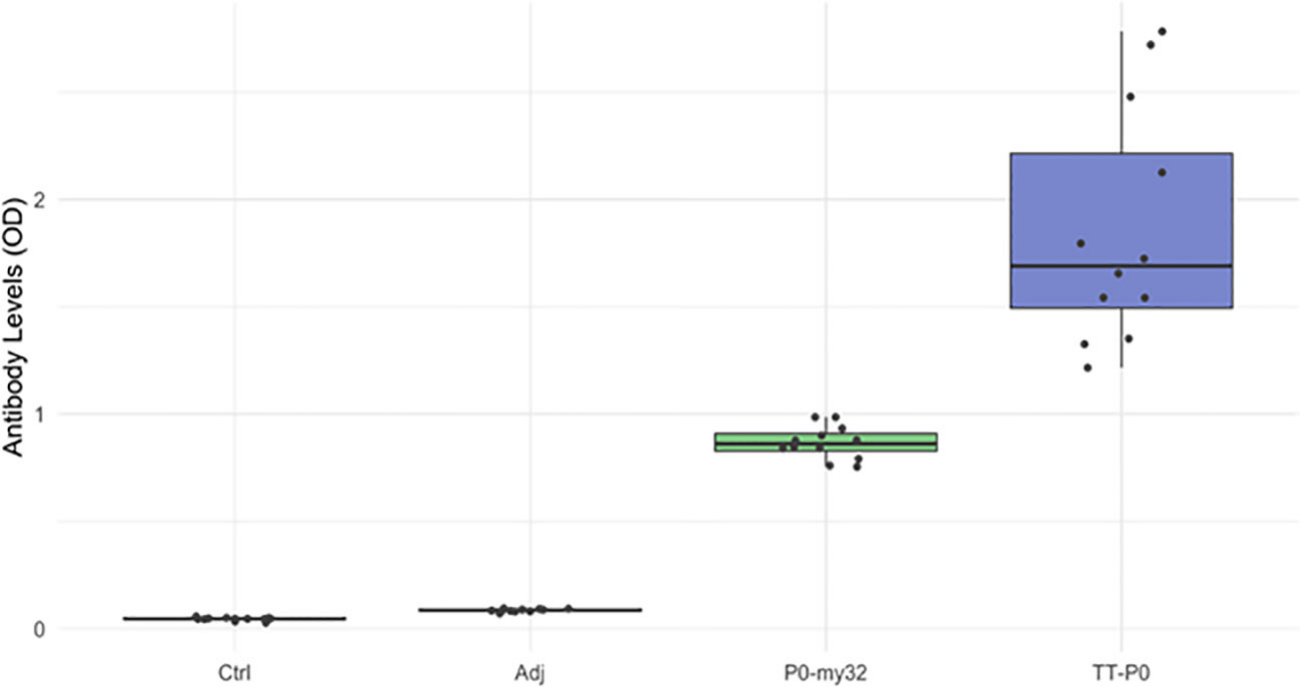
Antibody levels (OD_492 nm_) in the vaccine and control groups (n=12 fish per group) at 12 months post-vaccination. Data are presented as 25/75 quartiles and 95% CI (spikes). TT-P0 group has significantly higher OD values than P0-my32 (p=0.0001). Ctrl: non-injected fish; Adj: injected with vehicle buffer formulated in Montanide ISA50 V2; P0-my32: injected with 0.1 mL of P0-my32 vaccine formulation (equivalent to 100 µg of P0-my32 antigen); TT-P0: Fish were injected with 0.1 mL of TT-P0 vaccine formulation (equivalent to 100 µg of TT-P0 antigen).

Plasma samples collected at final counting (12 months post boost) were assessed for the level of circulating antibody against homologous antigens by ELISA (**Figure 7**). The TT-P0 vaccinated fish had the highest OD values, more than twice the value found for P0-my32 (p=0.0001), which indicates a higher amount of specific circulating antibodies. We were not able to detect antibody response in the adjuvant and non-injected control groups because these groups had OD values at the background level.

We also plotted antibody responses against lice numbers for all groups. Still, we found no clear trend (**Supplementary Figure S8**), apart from a tendency for lice numbers to decline with increasing OD values in the TT-P0 group.

## 4 Discussion

Vaccines developed using modern approaches have shown great potential for the upcoming aquaculture vaccines and they are an alternative for traditional fish vaccines (22–24). These modern vaccine approaches has targeted specific pathogen components. For example, very recently a peroxiredoxin-2-derived peptide of 13 aa was selected for salmon immunization against sea lice after high-throughput proteomic analyses of blood samples from Atlantic salmon with a high lice infestation and bioinformatics analysis (25).

The main finding of present study is that the P0 protein fused with the TCEs from tetanus toxin and measles virus at the N-terminus peptide elicits a significant reduction of lice infestation for up to 12 months post-vaccination. The P0 protein has two well-known biological functions. First, it is an essential component of ribosomes in assembling the 60S ribosomal unit and the cell synthesis machinery (26). Secondly, it plays a role in DNA repair and apoptosis when dephosphorylated. P0 is localized intracellularly in cell membranes and saliva, but the functions associated with the latter localization are still unknown (27–30).

My32/akirin proteins are involved in a wide range of processes through direct or indirect regulation of gene transcription and are downstream components of immune deficiency (IMD) pathway in arthropods (31). My32 gene knockdown affected growth, fertility, molting, survival, weight, and oviposition in both, ticks and sea lice (14, 15, 32). These proteins are present in all development stages of lice (14, 15), which makes them potentially good targets for immunological control of sea lice infestation.

Both antigens have been previously tested for their potential to control sea lice infestations in salmon (14, 17). Still, the effect of combining P0 and my32 in a chimeric protein or the long-term protection has just been studied. TT-P0 vaccinated fish have significantly fewer lice (moveable stages) than the adjuvant and control groups for all three challenges. P0-my32 vaccinated fish also had significantly lower infestation after vaccination, but the effect waned earlier than in the TT-P0 group. P0-my32 vaccinated fish also had lower circulating antibody levels at 12 months post-vaccination than the TT-P0 group and had significantly higher side-effect scores at this time point than the TT-P0 group. Accordingly to these results, TT-P0 vaccine candidate seems to be a better choice to advance in a therapeutic vaccine development against sea lice. Fish in the adjuvant control group had fewer parasites per fish than non-vaccinated controls which indicate a stimulation of innate defenses triggered by the adjuvant Montanide™ ISA50 V2. This adjuvant has also been used previously in salmon to test sea lice vaccine candidates (17, 33). The fact that the oil adjuvant upregulates innate immune responses at early times post-immunization in Atlantic salmon has been shown in different fish species (34–36). Limited studies have shown that oil adjuvants induce a pro-inflammatory response in fish and this response can play a role in reducing lice infestation (37).

There are relatively few studies on vaccination against *L. salmonis* infestation in salmon (17, 19, 25, 33). Therefore, there needs to be more scientific background to build an understanding of protective immune mechanisms. On the other hand, host immune responses to *L. salmonis* infestation in non-vaccinated (naïve) Atlantic salmon have been the subject of several studies. It has been reasonably well documented that infestation will modulate local and systemic innate and adaptive immune responses, primarily based on differential regulation of gene expression. In contrast, underlying functional immune mechanisms have not been studied (38–41). Naïve susceptible salmon are typically biased toward Th2 responses (tissue preservation) (38, 42). At the same time, the immune response in Atlantic salmon families associated with sea lice resistance have been linked to an immune response with a Th1 bias (37). Studies that include active immunization of salmon before challenge with copepodids have found modulation of pro-inflammatory responses with differential expression of Th1 and Th2 cytokines linked to reduced infestation (17). However, no clear pattern or profile of a protective response has been shown. It should also be added that the effects of lowering lice numbers have been moderate (17). Others have demonstrated strong humoral immune responses towards recombinant lice proteins that are related to midgut function and blood digestion in lice, with moderate-to-good reduction of infestation for adult females (13-29%) or male lice (27-50%) (33). No clear correlation was found between high circulating antibody levels and reduced lice infestation (33).

We found that the best-protected group had the highest homologous circulating antibody levels, but to what extent antibodies play a role in protecting skin mucosa during lice infestation remains to be proven. Following immunization with TT-P0/TCE proteins, the strong antibody response aligns with previous studies (16). Similar to other studies, we did not find a clear correlation between the level of circulating antibodies and lice numbers (33) but we had a limited number of plasma samples from the different groups and only one sampling time. Thus, further experiments increasing the number of samples and sampling times, could provide more evidences about the correlation between antibody and infestation levels. Additionally, the secondary antibody used in the ELISA setup detects both IgM and IgT, where IgT is primarily found in the mucosal lining (43). However, the extent to which it would contribute to reducing the number of attached lice is unknown.

Currently, no functional assays are available to measure or quantify T-cell responses in salmon, and transcript profiling is used. We did not include gene expression studies in this experiment as it has previously been shown that the promiscuous T cell epitopes of tetanus toxin/measles protein (TCEs) conjugated to TT-P0 facilitate a mixed Th1/Th2/T17/Treg pattern in salmon (17). In hindsight, and the lack of correlation between circulating antibodies and lice infestation, this could have shed more light on underlying immune mechanisms.

More recently, a peptide-based vaccine based on the P0 acidic ribosomal protein of ticks (residues 282 to 301 (pP0)) and conjugated to keyhole limpet hemocyanin (KLH) of gastropod *Megathura crenulata* or Bm86 was evaluated for its ability to impact ticks viability in *Rhipicephalus* spp (44–46). and *Amblyomma mixtum* (47). These vaccine candidates induced high immunogenicity and in general they have a good efficacy. On the contrary, when the same strategy was employed in the fish *Labeo rohita* against the parasite *Argulus siamensis*, only a partial protection was achieved. These differential results emphasize the importance of antigen design and delivery that must be adjusted to the specific host and pathogen (48).

Irrespective of the underlying mechanisms, the reported percent of reduction in parasite number is the highest and long term reported in laboratory-controlled conditions. Previous laboratory trials employing my32, TLR-6, or a potassium chloride and amino acid transporter as antigens gave reductions in parasite numbers ranging from 31 to 57%, either at the chalimus or adult stage or in the case of my32 only at the second parasite generation (14, 33). Later studies using the peroxiredoxin-2 peptide-derived vaccine demonstrated a 60–70% protection against early-stage *L. salmonis* experimental infection and 92% reduction in the number of adult lice in *C. rogercresseyi* but only for a short period of time and with significant loss of lice throughout the experiment (25).

The results obtained in this study for the TT-P0/TCE antigen are superior to those obtained in previous studies (17). The main difference in the procedure used herein as compared to the previous study is the prime-boost strategy with homologous antigens (and formulations) in freshwater. In contrast, a booster in seawater was used in the previous survey. Here, we also show the duration of protection, equivalent to (almost) the entire salmon production cycle in a commercial setting.

Two approaches for challenges were used; the first two were performed with vaccine groups kept in individual tanks (in triplicate), with a common-garden setup for the final challenge. Triplicate tanks (per group) allowed compensation for the so-called “tank effect,” and low inter-tank variation of lice numbers was observed (S.E.M<0.4 for average lice numbers for all the groups). A 20% reduction in infestation in the TT-P0 vaccinated group at the last challenge using a standard garden setup is intriguing and warrants further studies, particularly since moveable lice stages can redistribute between fish in the tank using this approach, thus mimicking a natural condition and this can potentially mask the effect of vaccination (33, 49) or it could be related to a waning immune response over time.

From studies of vaccination against ticks in mammals, control and reduction of the population is possible by combined control programs with integrated management strategies, where vaccination constitutes a vital pillar, even when vaccine efficacy is less than 100% (10, 46). Gavac^®^ is the cuban commercial vaccine against ticks based on Bm86 protein. It has been applied to more than 3 million cattle in Cuba, Venezuela, Mexico, Brazil, and Colombia with an efficacy ranging between 51% and 99%, depending on the tick strain, providing good documentation that immunologic control of tick infestation under field conditions is possible (10, 50–53). In this context, lice reductions obtained in laboratory conditions could be promising, particularly when combined with other control strategies.

In summary, the results of this study at the laboratory scale constitute the bases for the development of further experiments under large-scale production settings. One of the main challenges for these vaccines is the perception that they will act like chemicals with an on/off effect. The commercial Bm86-based Gavac^®^ vaccine has shown a reduced tick population in the context of an integrated control program. Its application reduced considerably the number of treatments with chemicals over time. Based on this experience, the reduction in the number of chemical delousing treatments over the salmon production cycle would be a viable approach to evaluate the impact of vaccination under field conditions.

## Data Availability

The original contributions presented in the study are included in the article/**Supplementary Material**, further inquiries can be directed to the corresponding author/s.

## References

[1] Parsons AE Escobar-Lux RH Hannisdal R Agnalt A-L Samuelsen OB. Anti-sea lice veterinary medicinal products on salmon farms: A review and analysis of their usage patterns, environmental fate and hazard potential. Rev Aquaculture. (2025) 17:e13006. doi: 10.1111/raq.13006

[2] Barret LT Oppedal F Robinson N Dempster T. Prevention not cure: a review of methods to avoid sea lice infestations in salmon aquaculture. Aquaculture Rev. (2020) 12:2527–43. doi: 10.1111/raq.12456

[3] Aaen SM Helgesen KO Bakke MJ Kaur K Horsberg TE. Drug resistance in sea lice: a threat to salmonid aquaculture. Trends Parasitol. (2015) 31:72–81. doi: 10.1016/j.pt.2014.12.006 25639521

[4] Burridge L Weis JS Cabello F Pizarro J Bostick K. Chemical use in salmon aquaculture: A review of current practices and possible environmental effects. Aquaculture. (2010) 306:7–23. doi: 10.1016/j.aquaculture.2010.05.020

[5] Overton K Dempster T Oppedal F Kristiansen TS Gismervik K Stien LH. Salmon lice treatments and salmon mortality in Norwegian aquaculture: a review. Rev Aquaculture. (2019) 11:1398–417. doi: 10.1111/raq.12299

[6] Walde CS Bang Jensen B Stormoen M Asche F Misund B Pettersen JM. The economic impact of decreased mortality and increased growth associated with preventing, replacing or improving current methods for delousing farmed Atlantic salmon in Norway. Prev Vet Med. (2023) 221:106062. doi: 10.1016/j.prevetmed.2023.106062 37939576

[7] Leclercq E Davie A Migaud H. Delousing efficiency of farmed ballan wrasse (Labrus bergylta) against *Lepeophtheirus salmonis* infecting Atlantic salmon (*Salmo salar*) post-smolts. Pest Manag Sci. (2014) 70:1274–82. doi: 10.1002/ps.2014.70.issue-8 24293262

[8] Imsland AKD Hanssen A Nytro AV Reynolds P Jonassen TM Hangstad TA. It works! Lumpfish can significantly lower sea lice infestation in large-scale salmon farming. Biol Open. (2018) 7:bio036301. doi: 10.1242/bio.036301 30177547 PMC6176945

[9] Bui S Geitung L Oppedal F Barrett LT. Salmon lice survive the straight shooter: A commercial scale sea cage trial of laser delousing. Preventive Veterinary Med. (2020) 181:105063. doi: 10.1016/j.prevetmed.2020.105063 32593083

[10] Suarez M Rubi J Pérez D Cordova V Salazar Y Vielma A. High impact and effectiveness of Gavac™ vaccine in the national program for control of bovine ticks Rhipicephalus microplus in Venezuela. Livestock Sci. (2016) 187:48–52. doi: 10.1016/j.livsci.2016.02.005

[11] Stutzer C Richards SA Ferreira M Baron S Maritz-Olivier C. Metazoan parasite vaccines: present status and future prospects. Front Cell Infect Microbiol. (2018) 8:67. doi: 10.3389/fcimb.2018.00067 29594064 PMC5859119

[12] Artigas-Jerónimo S Villar M Cabezas-Cruz A Valdés JJ Estrada-Peña A Alberdi A. Functional evolution of subolesin/akirin. Front Physiol. (2018) 9:1612. doi: 10.3389/fphys.2018.01612 30542290 PMC6277881

[13] de la Fuente J Moreno-Cid JA Galindo RC Almazan C Kocan KM Merino O. Subolesin/Akirin vaccines for the control of arthropod vectors and vectorborne pathogens. Transbound Emerg Dis. (2013) 60 Suppl 2:172–8. doi: 10.1111/tbed.12146 24589118

[14] Carpio Y Basabe L Acosta J Rodriguez A Mendoza A Lisperger A. Novel gene isolated from *Caligus rogercresseyi*: a promising target for vaccine development against sea lice. Vaccine. (2011) 29:2810–20. doi: 10.1016/j.vaccine.2011.01.109 21320542

[15] Carpio Y Garcia C Pons T Haussmann D Rodriguez-Ramos T Basabe L. Akirins in sea lice: first steps towards a deeper understanding. Exp Parasitol. (2013) 135:188–99. doi: 10.1016/j.exppara.2013.06.018 23850998

[16] Leal Y Velazquez J Hernandez L Swain JK Rodriguez AR Martinez R. Promiscuous T cell epitopes boosts specific IgM immune response against a P0 peptide antigen from sea lice in different teleost species. Fish Shellfish Immunol. (2019) 92:322–30. doi: 10.1016/j.fsi.2019.06.018 31200071

[17] Swain JK Carpio Y Johansen LH Velazquez J Hernandez L Leal Y. Impact of a candidate vaccine on the dynamics of salmon lice (*Lepeophtheirus salmonis*) infestation and immune response in Atlantic salmon (*Salmo salar* L.). PLoS One. (2020) 15:e0239827. doi: 10.1371/journal.pone.0239827 33006991 PMC7531828

[18] Studier FW. Protein production by auto-induction in high density shaking cultures. Protein Expr Purif. (2005) 41:207–34. doi: 10.1016/j.pep.2005.01.016 15915565

[19] Gislefoss E Abdelrahim Gamil AA Øvergard AC Evensen O. Identification and characterization of two salmon louse heme peroxidases and their potential as vaccine antigens. iScience. (2023) 26:107991. doi: 10.1016/j.isci.2023.107991 37854698 PMC10579435

[20] Midtlyng PJ Reitan LJ Speilberg L. Experimental studies on the efficacy and side-effects of intraperitoneal vaccination of Atlantic salmon (*Salmo salar* L.) against furunculosis. Fish Shellfish Immunol. (1996) 6:335–50. doi: 10.1006/fsim.1996.0034

[21] Faul F Erdfelder E Lang A-G Buchner A. G*Power 3: A flexible statistical power analysis program for the social, behavioral, and biomedical sciences. Behav Res Methods. (2007) 39:175–91. doi: 10.3758/BF03193146 17695343

[22] Ma J Bruce TJ Jones EM Cain KD. A review of fish vaccine development strategies: conventional methods and modern biotechnological approaches. Microorganisms. (2019) 7:569. doi: 10.3390/microorganisms7110569 31744151 PMC6920890

[23] Mondal H Thomas J. A review of the recent advances and application of vaccines against fish pathogens in aquaculture. Aquacult Int. (2022) 30:1971–2000. doi: 10.1007/s10499-022-00884-w PMC9059915 35528247

[24] Jose Priya TA Kappalli S. Modern biotechnological strategies for vaccine development in aquaculture – Prospects and challenges. Vaccine. (2022) 40:5873–81. doi: 10.1016/j.vaccine.2022.08.075 36088192

[25] Johny A Ilardi P Olsen RE Egelandsdal B Slinde E. A Proof-of-Concept Study to Develop a Peptide-Based Vaccine against Salmon Lice Infestation in Atlantic Salmon (*Salmo salar* L.). Vaccines. (2024) 12:456. doi: 10.3390/vaccines12050456 38793707 PMC11125789

[26] Santos C Ballesta JPG Ribosomal Protein PO. Contrary to phosphoproteins PI and P2, is required for ribosome activity and saccharomyces cerevisiae viability. J Biol Chem. (1994) 269, 22:15689–96. doi: 10.1016/S0021-9258(17)40736-8 8195220

[27] Rodriguez-Gabriel MA Remacha M Ballesta JP. The RNA interacting domain but not the protein interacting domain is highly conserved in ribosomal protein P0. J Biol Chem. (2000) 275:2130–6. doi: 10.1074/jbc.275.3.2130 10636918

[28] Singh S Sehgal A Waghmare S Chakraborty T Goswami A Sharma S. Surface expression of the conserved ribosomal protein P0 on parasite and other cells. Mol Biochem Parasitol. (2002) 119:121–4. doi: 10.1016/S0166-6851(01)00394-2 11755193

[29] Radulovic ZM Kim TK Porter LM Sze SH Lewis L Mulenga A. A 24–48 h fed *Amblyomma americanum* tick saliva immuno-proteome. BMC Genomics. (2014) 15:518. doi: 10.1186/1471-2164-15-518 24962723 PMC4099483

[30] Tirloni L Reck J Terra RM Martins JR Mulenga A Sherman NE. Proteomic analysis of cattle tick *Rhipicephalus (Boophilus) microplus* saliva: a comparison between partially and fully engorged females. PLoS One. (2014) 9:e94831. doi: 10.1371/journal.pone.0094831 24762651 PMC3998978

[31] Goto A Matsushita K Gesellchen V Chamy LE Kuttenkeuler D Takeuchi O. Akirins are highly conserved nuclear proteins required for NF-κB-dependent gene expression in drosophila and mice. Nat Immunol. (2008) 9:97–104. doi: 10.1038/ni1543 18066067 PMC2680477

[32] de la Fuente J Almazan C Blas-MaChado U Naranjo V Mangold AJ Blouin EF. The tick protective antigen, 4D8, is a conserved protein involved in modulation of tick blood ingestion and reproduction. Vaccine. (2006) 24:4082–95. doi: 10.1016/j.vaccine.2006.02.046 16580098

[33] Contreras M Karlsen M Villar M Olsen RH Leknes LM Furevik A. Vaccination with ectoparasite proteins involved in midgut function and blood digestion reduces salmon louse infestations. Vaccines (Basel). (2020) 8(1):32. doi: 10.3390/vaccines8010032 31963779 PMC7157638

[34] Mutoloki S Alexandersen S Evensen Ø. Sequential study of antigen persistence and concomitant inflammatory reactions relative to side-effects and growth of Atlantic salmon (Salmo salar L.) following intraperitoneal injection with oil-adjuvanted vaccines. Fish Shellfish Immunol. (2004) 16:633–44. doi: 10.1016/j.fsi.2003.10.002 15110337

[35] Mutoloki S Alexandersen S Gravningen K Evensen O. Time-course study of injection site inflammatory reactions following intraperitoneal injection of Atlantic cod (*Gadus morhua* L.) with oil-adjuvanted vaccines. Fish Shellfish Immunol. (2008) 24:386–93. doi: 10.1016/j.fsi.2007.08.009 18282765

[36] Mutoloki S Brudeseth B Reite OB Evensen O. The contribution of Aeromonas salmonicida extracellular products to the induction of inflammation in Atlantic salmon (*Salmo salar* L.) following vaccination with oil-based vaccines. Fish Shellfish Immunol. (2006) 20:1–11. doi: 10.1016/j.fsi.2005.01.005 16018934

[37] Holm H Santi N Kjøglum S Perisic N Skugor S Evensen Ø. Difference in skin immune responses to infection with salmon louse (*Lepeophtheirus salmonis*) in Atlantic salmon (*Salmo salar* L.) of families selected for resistance and susceptibility. Fish Shellfish Immunol. (2015) 42:384–94. doi: 10.1016/j.fsi.2014.10.038 25449368

[38] Skugor S Glover KA Nilsen F Krasnov A. Local and systemic gene expression responses of Atlantic salmon (*Salmo salar* L.) to infection with the salmon louse (*Lepeophtheirus salmonis*). BMC Genomics. (2008) 9:498. doi: 10.1186/1471-2164-9-498 18945374 PMC2582245

[39] Tadiso TM Krasnov A Skugor S Afanasyev S Hordvik I Nilsen F. Gene expression analyses of immune responses in Atlantic salmon during early stages of infection by salmon louse (*Lepeophtheirus salmonis*) revealed bi-phasic responses coinciding with the copepod-chalimus transition. BMC Genomics. (2011) 12:141. doi: 10.1186/1471-2164-12-141 21385383 PMC3062619

[40] Braden LM Koop BF Jones SR. Signatures of resistance to Lepeophtheirus salmonis include a TH2-type response at the louse-salmon interface. Dev Comp Immunol. (2015) 48:178–91. doi: 10.1016/j.dci.2014.09.015 25453579

[41] Fast MD Muise DM Easy RE Ross NW Johnson SC. The effects of Lepeophtheirus salmonis infections on the stress response and immunological status of Atlantic salmon (*Salmo salar*). Fish Shellfish Immunol. (2006) 21:228–41. doi: 10.1016/j.fsi.2005.11.010 16483797

[42] Jodaa Holm H Wadsworth S Bjelland AK Krasnov A Evensen Ø Skugor S. Dietary phytochemicals modulate skin gene expression profiles and result in reduced lice counts after experimental infection in Atlantic salmon. Parasit Vectors. (2016) 9:271. doi: 10.1186/s13071-016-1537-y 27164990 PMC4862074

[43] Zhang YA Salinas I Li J Parra D Bjork S Xu Z. IgT, a primitive immunoglobulin class specialized in mucosal immunity. Nat Immunol. (2010) 11:827–35. doi: 10.1038/ni.1913 PMC3459821 20676094

[44] Rodriguez-Mallon A Encinosa PE Mendez-Perez L Bello Y Rodriguez Fernandez R Garay H. High efficacy of a 20 amino acid peptide of the acidic ribosomal protein P0 against the cattle tick, *Rhipicephalus microplus* . Ticks Tick Borne Dis. (2015) 6:530–7. doi: 10.1016/j.ttbdis.2015.04.007 25958782

[45] Rodriguez-Mallon A Fernandez E Encinosa PE Bello Y Mendez-Perez L Ruiz LC. A novel tick antigen shows high vaccine efficacy against the dog tick, *Rhipicephalus sanguineus* . Vaccine. (2012) 30:1782–9. doi: 10.1016/j.vaccine.2012.01.011 22245603

[46] Rodriguez Mallon A Javier Gonzalez L Encinosa Guzman PE Bechara GH Sanches GS Pousa S. Functional and mass spectrometric evaluation of an anti-Tick antigen based on the P0 peptide conjugated to bm86 protein. Pathogens. (2020) 9(6):513. doi: 10.3390/pathogens9060513 32630414 PMC7350365

[47] Rodriguez-Mallon A Encinosa Guzman PE Bello Soto Y Rosales Perdomo K Montero Espinosa C Vargas M. A chemical conjugate of the tick P0 peptide is efficacious against *Amblyomma mixtum* . Transbound Emerg Dis. (2020) 67 Suppl 2:175–7. doi: 10.1111/tbed.13455 31975511

[48] Kar B Mohapatra A Mohanty J Sahoo PK. Evaluation of ribosomal P0 peptide as a vaccine candidate against *Argulus siamensis* in *Labeo rohita* . Open Life Sci. (2017) 12:99–108. doi: 10.1515/biol-2017-0011

[49] Ritchie G. The host transfer ability of *Lepeophtheirus salmonis* (Copepoda: Caligidae) from farmed Atlantic salmon, *Salmo salar* L. J Fish Dis. (1997) 20:153–7. doi: 10.1046/j.1365-2761.1997.00285.x

[50] Canales M Enriquez A Ramos E Cabrera D Dandie H Soto A. Large-scale production in *Pichia pastoris* of the recombinant vaccine Gavac against cattle tick. Vaccine. (1997) 15:414–22. doi: 10.1016/S0264-410X(96)00192-2 9141213

[51] de la Fuente J Rodriguez M Montero C Redondo M Garcia-Garcia JC Mendez L. Vaccination against ticks (*Boophilus* spp.): the experience with the Bm86-based vaccine Gavac. Genet Anal. (1999) 15:143–8. doi: 10.1016/S1050-3862(99)00018-2 10596754

[52] de la Fuente J Rodriguez M Redondo M Montero C Garcia-Garcia JC Mendez L. Field studies and cost-effectiveness analysis of vaccination with Gavac against the cattle tick. Boophilus microplus. Vaccine. (1998) 16:366–73. doi: 10.1016/s0264-410x(97)00208-9 9607057

[53] Valle MR Mèndez L Valdez M Redondo M Espinosa CM Vargas M. Integrated control of *Boophilus microplus* ticks in Cuba based on vaccination with the anti-tick vaccine Gavac TM. Exp Appl Acarology. (2004) 34:375–82. doi: 10.1007/s10493-004-1389-6 15651533

