# Supplementary material for: Prime-boost vaccination with chimeric antigens adjuvanted in Montanide™ ISA50 V2 confers protection against experimental Lepeophtheirus salmonis infestation in Atlantic salmon (Salmo salar L.)
Source: Front Immunol. 2025 May 21;16:1570948. doi: 10.3389/fimmu.2025.1570948 (PMC12133460; doi:10.3389/fimmu.2025.1570948)
Supplement: Supplementary file 1 [file DataSheet1.pdf]

Supplementary Files

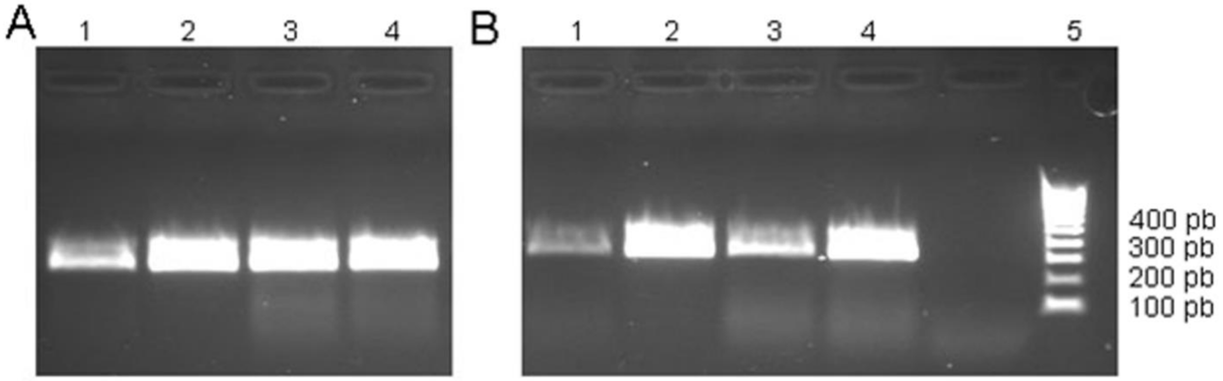

Supplementary figure S1. Expression analysis by RT-PCR of (A) β actin and (B) P0 mRNAs in the different *L. salmonis* developmental stages. Agarose gel electrophoresis 2% (p/v) of a representative PCR reaction per developmental stage. Lane 1: nauplius, lane 2: copepodids, lane 3: chalimus I–IV, lane 4: adults, lane 5: molecular weight marker.

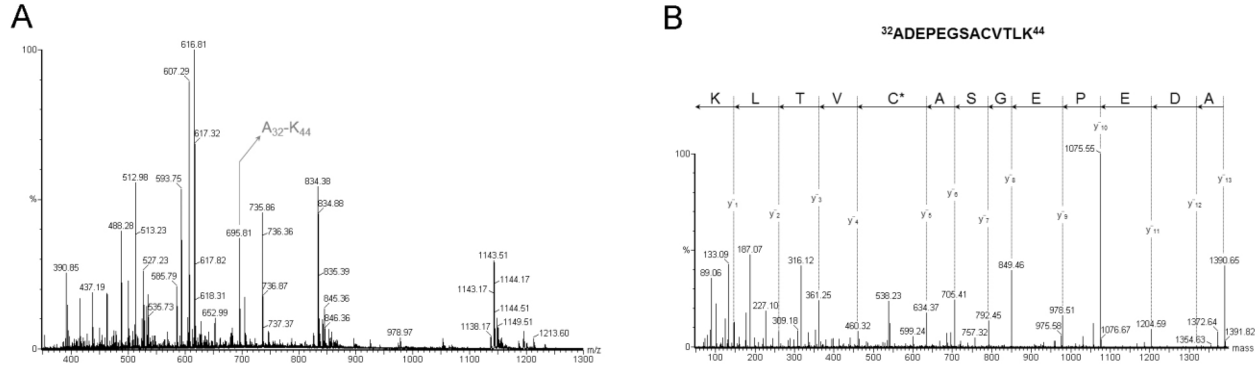

Supplementary figure S2. Identification by mass spectrometry of the recombinant protein P0-my32. (A) ESI-MS spectrum of the peptides obtained from the trypsin digestion of the gel band corresponding to the purified P0-my32 protein. (B) Spectrum ESI-MS / MS m / z 695.81.

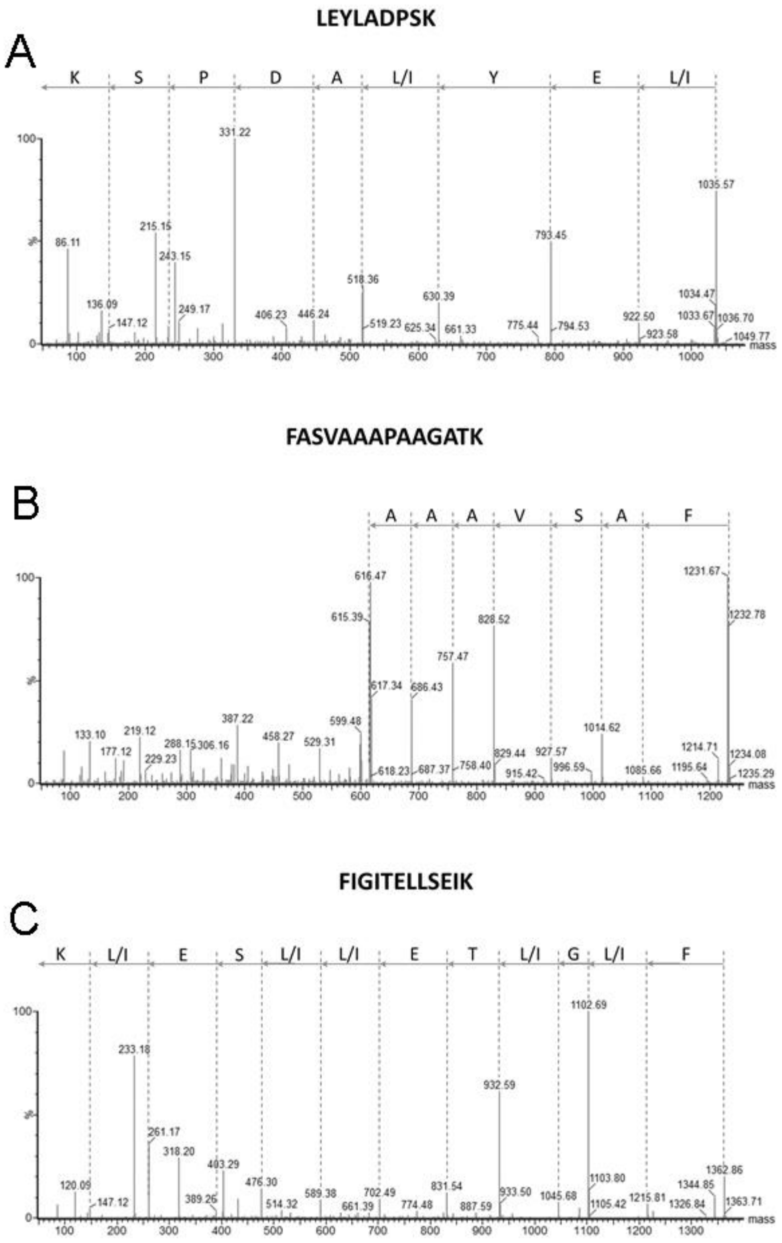

Supplementary figure S3. The recombinant protein TT-P0 was identified by mass spectrometry. (A-C) Peptide sequences obtained.

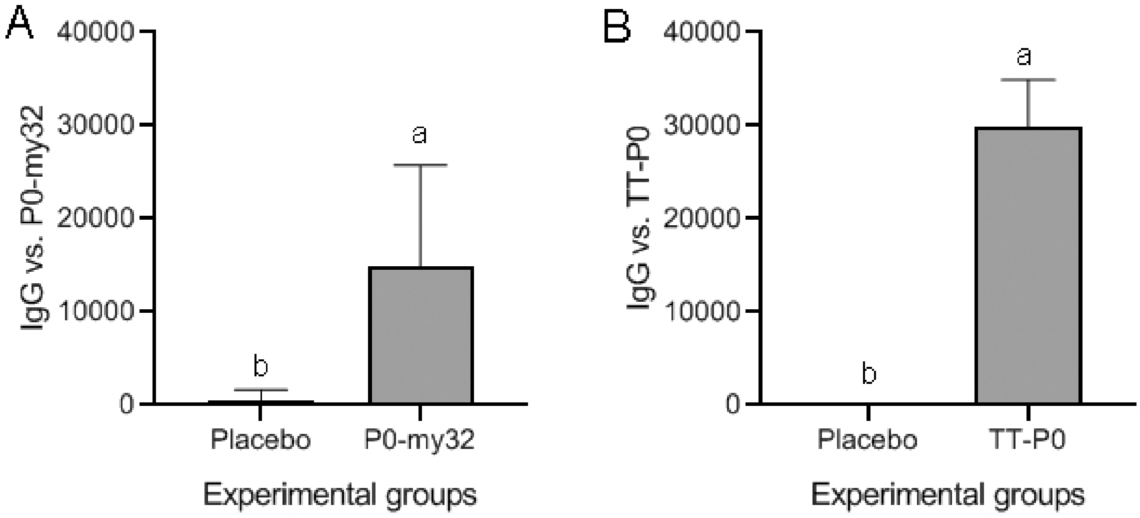

Supplementary figure S4. Evaluation of immunogenicity in mice: (A) P0-my32 and (B) TT-P0 vaccine formulations. Ten mice per group were immunized by subcutaneous injection with P0-my32-Ls or TT-P0 vaccine formulations at 20 µg per mouse in a total volume of 20 µL on days 0 and 14. Control mice were immunized with the same volume of vehicle buffer formulated in Montanide ISA50 V2. Blood was collected by retro-orbital bleeding 28 after the beginning of the experiment. ELISA analyzed specific IgG antibody titers against P0-my32 or TT-P0 recombinant proteins. The titer was the highest dilution, giving an optical density twice the value of the pre-immune serum. Different letters represent statistical differences determined by the Wilcoxon range test ($p<0.01$). Data are presented as geometric mean with 95% confidence intervals.

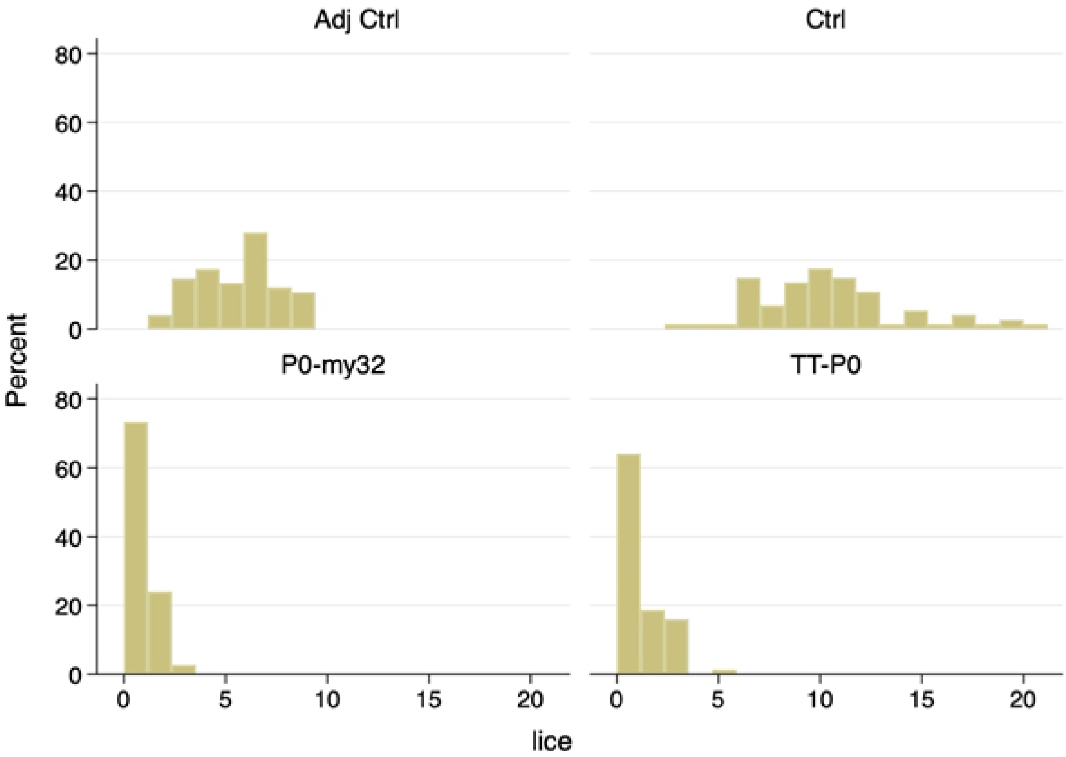

Supplementary Figure S5. Histogram showing the distribution of lice numbers in different groups at 32 days after the first challenge. Y-axis represents the percentage of fish with the number of lice indicated in x-axis. P0-my32 and TT-P0 are right skewed, with a high proportion of fish having 0 or 1 louse/per fish. Adj ctrl: injected with vehicle buffer formulated in Montanide ISA50 V2; Ctrl: non-injected fish; P0-my32: Fish were injected with 0.1 mL of P0-my32 vaccine formulation (equivalent to 100 µg of P0-my32 antigen); TT-P0: Fish were injected with 0.1 mL of TT-P0 vaccine formulation (equivalent to 100 µg of TT-P0 antigen).

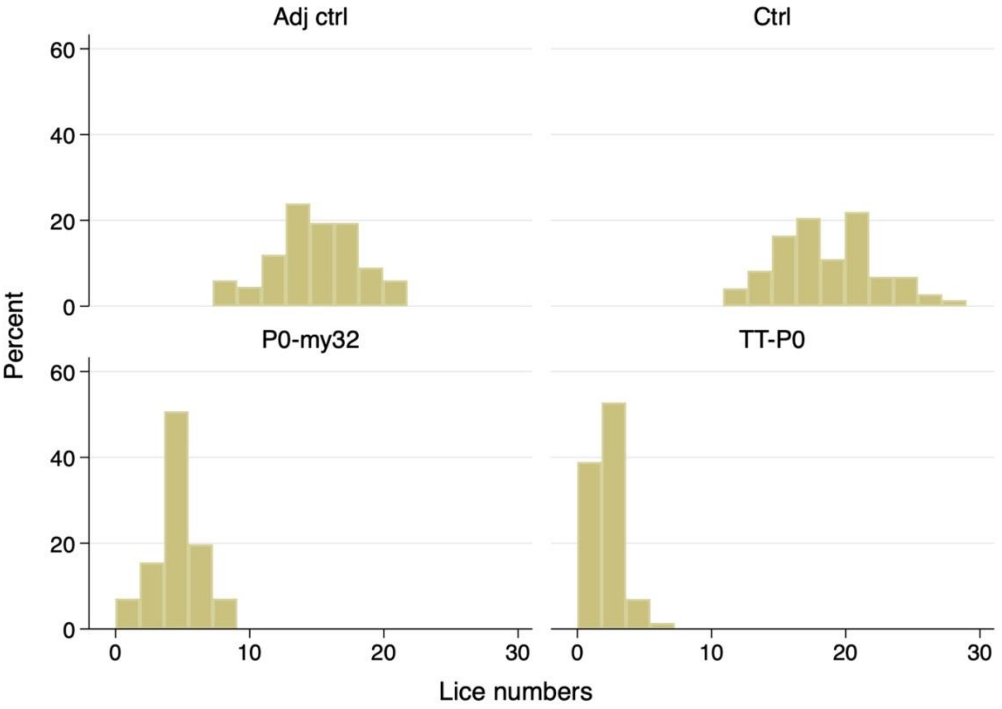

Supplementary Figure S6. Histogram showing the distribution of lice numbers in different groups at 40 days post the second challenge. Y-axis represents the percentage of fish with the number of lice indicated in x-axis. The distribution is closer to normal for all groups, with the TT-P0 group still showing right skewing. Adj ctrl: injected with vehicle buffer formulated in Montanide ISA50 V2; Ctrl: non-injected fish; P0-my32: Fish were injected with 0.1 mL of P0-my32 vaccine formulation (equivalent to 100 µg of P0-my32 antigen); TT-P0: Fish were injected with 0.1 mL of TT-P0 vaccine formulation (equivalent to 100 µg of TT-P0 antigen).

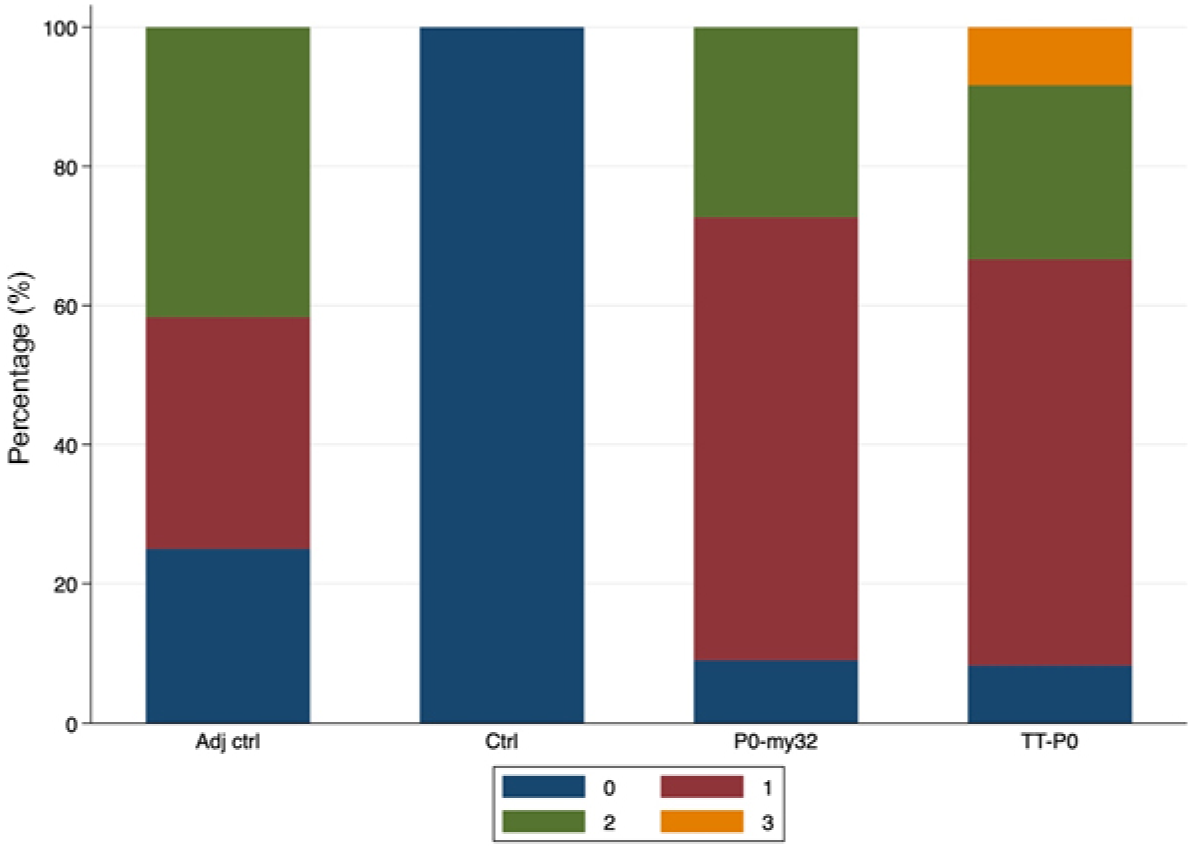

Supplementary Figure S7. Melanin scores assigned for the experimental groups. Melanin deposition was recorded at the final counting at the site of injection and in the visceral peritoneum (over the internal organs) and scored from 0 to 3. The percentage (%) distribution by score is given on the y-axis for the different groups (x-axis). Adj ctrl: injected with vehicle buffer formulated in Montanide ISA50 V2; Ctrl: non-injected fish; P0-my32: Fish were injected with 0.1 mL of P0-my32 vaccine formulation (equivalent to 100 µg of P0-my32 antigen); TT-P0: Fish were injected with 0.1 mL of TT-P0 vaccine formulation (equivalent to 100 µg of TT-P0 antigen).

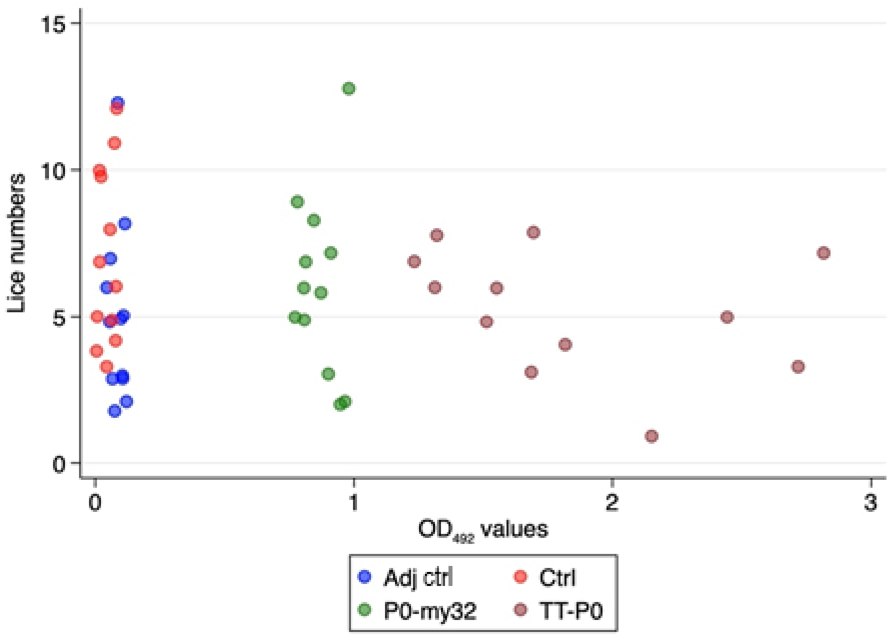

Supplementary Figure S8. OD_{492} values for individual fish were plotted against lice numbers for the different groups. TT-P0 group has significantly higher OD values than P0-my32 (p=0.0001). Adj ctrl: injected with vehicle buffer formulated in Montanide ISA50 V2; Ctrl: non-injected fish; P0-my32: Fish were injected with 0.1 mL of P0-my32 vaccine formulation (equivalent to 100 µg of P0-my32 antigen); TT-P0: Fish were injected with 0.1 mL of TT-P0 vaccine formulation (equivalent to 100 µg of TT-P0 antigen).

Supplementary Table S1. Environmental parameters and handling during vaccination/ challenges experiment with P0-my32 and TT-P0 antigens in *Salmo salar*

**Salinity**	33-35‰ before transfer/challenge.
**Stocking density**	Max 40 Kg/ m^3
**Temperature**	13-14 °C ± 1°C during immunization (in freshwater)<br>10-11 °C during challenge (in sea water). Last challenge was conducted at water temperature of 12-13 °C.
**Flow**	1.2-2.2 L/Kg/min
**Water discharge**	Tube overflow system
**Cleaning**	Once a day
**Feeding**	Excess feeding by feeding automats
